# Editorial: New molecular pathways in thyroid biology: role of coding and noncoding genes in thyroid pathophysiology, volume II

**DOI:** 10.3389/fendo.2025.1729485

**Published:** 2025-10-30

**Authors:** Cesar Seigi Fuziwara, Juan Pablo Nicola

**Affiliations:** ^1^ Department of Cell and Developmental Biology, Institute of Biomedical Sciences, University of São Paulo, São Paulo, Brazil; ^2^ Department of Biochemistry, Federal University of Sao Paulo, Sao Paulo, Brazil; ^3^ Department of Clinical Biochemistry (CIBICI-CONICET), Faculty of Chemical Sciences, National University of Córdoba, Córdoba, Argentina; ^4^ Clinical Biochemistry and Immunology Research Center, National Scientific and Technical Research Council (CIBICI-CONICET), Córdoba, Argentina

**Keywords:** thyroid cancer (THCA), tumor microenvironment - TME, metastasis, circRNA, PANoptosis, FNA (fine needle aspiration), molecular analyses

In Volume II of the Research Topic “*New molecular pathways in thyroid cancer and pathophysiology: Role of coding and noncoding genes*,” we continued to explore recent advances in the understanding of thyroid biology, especially those involved in thyroid oncogenesis and progression.

Thyroid cancer is often a curable disease detected predominantly as papillary thyroid cancer (PTC), the most common variant: it is classified as a well-differentiated tumor (DTC) that maintains a certain similarity to a normal thyroid gland, mainly the ability to concentrate iodine. However, a small fraction of PTC cases may exhibit more aggressive behavior and an enhanced probability of recurrence, often resulting in poor outcomes. In this context, Liu et al. investigated how the molecular genetic background of DTC could help predict the risk of recurrence and reported that high-risk DTC often exhibits late-hit genetic alteration. Moreover, Liu et al. analyzed the differential expression of high- and low-risk dedifferentiation PTC and found that, besides the thyroid differentiation score (TDS), the signatures of 17 differentially expressed genes (DEGs) were associated with dedifferentiation and that the complement pathway was a key component, with CD55 being overactivated in the high-risk group and tall-cell PTC.

In the process of cancer progression, metastasis dissemination is an indicator of recurrence and poor prognosis, as tumor cells escape from primary sites and acquire more migratory and invasive characteristics. Hu et al. investigated the differential expression of metastatic papillary microcarcinoma by RNAseq and identified the increase in thrombospondin 4 (THBS4) as a potential new biomarker for predicting lymph node metastasis dissemination, which correlates with the presence of PDGFRA+ inflammatory cancer-associated fibroblasts in the tumor. Additionally, Wang et al. showed PMAIP1/NOXA is a new player in follicular thyroid cancer (FTC) progression and metastasis by inducing the transcription factor FOSL3 via the Wnt signaling pathway and contributing to cell migration and invasion of FTC.

Using an extensive strategy irrespective of genetic background or variant, Zou et al. investigated a common intersection in the transcriptome among different variants of metastatic thyroid cancer derived from transgenic mouse models and found a signature that points to immune cell microenvironment modulation. In this environment, the metastatic cells produce more inflammatory cytokines and chemokines, such as IL6 and IL6R, and express the immunomodulatory PDL1. Additionally, Ma et al. investigated the impact of Hashimoto thyroiditis (HT) on the PTC microenvironment using single-cell RNA sequencing analysis and identified a tumor microenvironment where immune and stromal cells are different from non-HT, creating a TSH-inhibiting environment for PTC growth.

Meanwhile, Li et al. investigated the expression of genes related to PANoptosis, a new type of cell death that combines key features of pyroptosis, apoptosis, and necroptosis, and identified the signatures of eight key genes, including the tumor necrosis factor receptor superfamily (TNFRSF) and PMAIP1 cited previously, linking inflammatory cell death to immune microenvironment dysregulation in thyroid cancer.

To identify new vulnerabilities for thyroid cancer cells, Ma et al. bring new aspects of thyroid cancer biology linked to the deregulation of circular RNAs (circRNAs), a class of noncoding RNAs with a covalent continuous closed loop. CircRNAs may act as competing endogenous RNAs (CeRNAs) that displace miRNA-mediated regulation at target genes, acting as miRNA sponges, thus modulating signaling pathways involved in tumorigenesis and cancer progression. Another interesting context of the role of noncoding RNA deregulation is shown by Chen et al., who reported a case report and literature review of thyroblastoma, a singular disease that presents distinctive primitive characteristics and shows prevalent mutations in *DICER1*, a key nuclease in the microRNA biogenesis pathway. Finally, correct diagnosis of thyroid cancer is essential for the assessment of prognosis and clinical practice. Wu et al. built a risk stratification model based on the FNA washout DNA copy number variation using low-coverage whole-genome sequencing to identify high-risk lymph node metastasis.

In sum, we hope the Research Topic “*New molecular pathways in thyroid cancer and pathophysiology: Role of coding and noncoding genes, Volume II*,” has contributed to accelerating the understanding of this thrilling field of thyroid biology and that further studies can bring fruitful applications for thyroid pathology treatment.

